# Urate Lowering Therapy with Febuxostat in Daily Practice—A Multicentre, Open-Label, Prospective Observational Study

**DOI:** 10.1155/2014/123105

**Published:** 2014-09-03

**Authors:** Anne-Kathrin Tausche, Monika Reuss-Borst, Ute Koch

**Affiliations:** ^1^Department of Rheumatology, University Clinic “Carl Gustav Carus”, Technical University Dresden, Fetscherstraße 74, 01307 Dresden, Germany; ^2^Rheumatology, Rehaklinik am Kurpark, Kurhausstraße 9, 97688 Bad Kissingen, Germany; ^3^Sybelstraße 42, 10629 Berlin, Germany

## Abstract

*Introduction.* Febuxostat, a novel xanthine oxidase inhibitor for the treatment of symptomatic hyperuricemia, showed superiority over allopurinol in the reduction of serum uric acid levels in pivotal studies. Whether this holds true the FORTE (febuxostat in the oral urate lowering treatment: effectiveness and safety) study was conducted to evaluate treatment with febuxostat under daily practice conditions. *Materials/Methods.* The multicentre, open-label, and prospective observational study was conducted in 1,690 German medical practices from 9/2010 to 5/2011. Safety and efficacy data were assessed at baseline and week 4. *Results.* Data from 5,592 gout patients (72.6% male, mean age 63.7 years) were collected. Under urate lowering treatment with febuxostat mean serum uric acid levels decreased significantly from 8.9 ± 1.9 mg/dL (534.0 ± 114.6 *μ*mol/L) at baseline to 6.2 ± 2.5 mg/dL (372.0 ± 150.0 *μ*mol/L) at week 4. 67% which reached the mean uric acid target (6.1 ± 1.0 mg/dL [366.0 ± 59.4 *μ*mol/L]). Only 43.1% of patients received concomitant flare prophylaxis. A total of 178 adverse events (mostly gout flares) were reported in 152 patients (2.6%). *Conclusion.* Febuxostat lowers serum uric acid levels effectively in routine clinical practice. Overall, treatment with febuxostat in both available dosages (80 mg/120 mg) was safe and well tolerated.

## 1. Introduction

Gout is the most common inflammatory arthritic disease. Gout incidence is increasing due to the aging population and concomitant rise in comorbidities, such as chronic renal impairment, as well as administration of drugs known to inhibit uric acid excretion (e.g., low-dose aspirin, thiazide, and loop diuretics) [1, 2]. A “modern” lifestyle (purine-rich diet, lack of physical exercise, and excessive alcohol consumption) and successively increased body mass index promote hyperuricaemia and gout [3, 4].

Persistent untreated hyperuricaemia can lead to depositions of monosodium urate in joints and soft tissues (known as tophi). The solubility of monosodium urate is strong temperature and pH dependency. The saturation threshold at 37°C is 6.8 mg/dL (~400 *μ*mol/L). Exceeding this threshold leads to crystallization.

Initially, characteristic flares occur as monoarticular arthritis, such as the classic podagra. During progression, other joints can be involved, leading to tophus formation and joint destruction. Only rarely have cases of tophus formation in interior organs, for example, valvular or pancreatic regions, been observed [5]. Other complications from gout include formation of kidney stones and deterioration of prevalent impaired renal function. Therefore gout is a systematic disease with complex metabolic and inflammatory correlations [5].

In the case of gout, medical intervention is indicated in order to prevent progression and to eliminate any tophi [4]. A sustained reduction of serum uric acid levels is crucial in this process. In Germany, due to the nonexistence of specific German guidelines regarding the treatment of gout, physicians may turn to the European League against Rheumatism (EULAR) for guidance [6, 7]. Based on current evidence, the EULAR recommends the reduction of serum uric acid (SUA) to levels below 6.0 mg/dL (≤360 *μ*mol/L) [6]. The British Society of Rheumatology recommends an even stricter target of <5 mg/dL (<300 *μ*mol/L) [8]. Recently published recommendations from the American College of Rheumatology (ACR) confirm both targets. The ACR recommends reducing SUA levels in the first instance to values below 6.0 mg/dL (360 *μ*mol/L). In the case of persisting symptoms (e.g., gout flares and palpable tophi) the reduction <5.0 mg/dL (300 *μ*mol/L) is recommended [9]. As, due to rapid dissolution of crystals, especially during initiation of potent urate lowering therapy gout flares can occur, the guidelines recommend also gout flare prophylaxis [6, 8, 10].

Since the 1960s, the uricostatic drug allopurinol as well as the uricosuric drugs benzbromarone and probenecid have been used to lower urate. Febuxostat has received marketing authorisation as a novel uricostatic drug in Germany in 2010. The available dosages are 80 mg and 120 mg. This novel xanthine oxidase inhibitor is indicated for the treatment of chronic hyperuricaemia in conditions where urate deposition has already occurred (including a history or presence of tophus and/or gouty arthritis) [11].

Unlike allopurinol, febuxostat is a nonpurine agent that inhibits xanthine oxidase competitively; however it is not degraded by the enzyme [12]. The pivotal clinical studies showed that significantly more febuxostat-treated patients met the EULAR recommended target of <6.0 mg/dL (<300 *μ*mol/L) compared to those receiving allopurinol 300 mg/day. The FACT study compared febuxostat 80 and 120 mg/day with allopurinol 300 mg/day over 1 year in 762 gout patients. In this study 53% and 62%, respectively, achieved the EULAR target compared to 21% of patients in the allopurinol group [13].

Considering that design and results of pivotal studies do not necessarily reflect the daily routine of practitioners, we conducted the first multicenter prospective observational study FORTE from September 2010 to May 2011. The aim of the study was to evaluate use, effectiveness, and safety of febuxostat in clinical practice routine.

## 2. Materials and Methods

### 2.1. Study Design

Effectiveness and safety of febuxostat in patients with gout were evaluated in a multicentre, open-label, and prospective observational study (FORTE, febuxostat in the oral urate lowering treatment: effectiveness and safety) conducted at 1,690 centres (general practitioners, internists) in Germany between September 2010 and May 2011. Observation time per patient was approximately 4 weeks. Safety and efficacy data were assessed at baseline and at week 4. The study was reviewed and approved by federal state law established Ethics Committees Counselling (TU Dresden: EK number 215072010). No diagnostic or therapeutic measures, exceeding the already necessary frame, were required for this study; treatment routine was not altered. All patients provided their written informed consent prior to entering the study. Patients were free to withdraw from the study at any time and for any reason.

### 2.2. Patients

Patients with confirmed diagnosis of gout (ICD10: M10.0∗) in whom medical urate lowering treatment with febuxostat was indicated according to their treating physicians were documented. In accordance with the participating investigator's assessment and based on the recommendations specified in the summary of products characteristics (SPC), febuxostat was administered over 4 weeks [11].

### 2.3. Documentation Parameters

Documentation was collected on standardized patient documentation forms. At baseline, data on patient demographics, gout manifestation, concomitant medication, and serum uric acid concentration was acquired. At the follow-up final visit, uric acid concentration after approximately 4 weeks of treatment was documented along with febuxostat dosage, flare prophylaxis, treatment assessment by the physician, and the planned future therapy (maintaining the initial dose/dose adjustment according to serum uric acid concentration or discontinuation). Adverse drug reactions (ADR) and serious adverse events (SAE) were documented on separate standardized documentation forms.

### 2.4. Primary Objectives

The primary objective of this observational study was to expand the knowledge base on the safety and tolerability, the reduction in serum uric acid levels, and the incidence of gout attacks during treatment period of 4 weeks with febuxostat under everyday conditions on a broad representative group of patients with chronic symptomatic hyperuricaemia.

### 2.5. Statistical Analyses

All patients with at least one administration of febuxostat were included in the statistical evaluation (ITT population). The collected data were analyzed with descriptive, epidemiological methods. Evaluation of the clinical course of uric acid levels was performed by intraindividual difference analysis (first versus last examination) using the Wilcoxon signed-rank test. All tests were two-sided, and significance was declared at the 0.05 level. The statistical analyses were performed by SIMW GmbH, Wegberg, Germany.

## 3. Results

### 3.1. Patient Disposition and Baseline Characteristics

In total, 5,948 patients with gout were enrolled from 1,690 German practices. 94.0% of patients (*n* = 5,592) were treated with febuxostat over the whole surveillance period (45.3 ± 36.4 days). All patients (*n* = 5,948) were included in the evaluation. Median time since gout diagnosis was 46 months (approximately 4 years) prior to the study. Within the previous 12 months patients had experienced between 2 and 3 gout flares. The majority of patients were male (72.6%). Mean age (±SD) was 63.7 ± 12.6 years.

Mean uric acid level at baseline was 8.9 ± 1.9 mg/dL (531.0 ± 114.6 *μ*mol/L).

75.5% of patients had at least one concomitant disease. Hypertension was prevalent in 69.0% of patients, hyperlipidemia in 43.0%, diabetes mellitus in 32.0%, and 16.4% had impaired renal function (eGFR < 89 mL/min). 3.3% of patients had a history of kidney stones.

Baseline demographics are summarized in Table 1.

### 3.2. Previous Treatment of Gout

At the time of first evaluation, of the 5,948 included patients, 80.31% (*n* = 4,777) received any urate lowering therapy (ULT), 19.69% were not on ULT or received only pain medication. Of the ULT treated patients 78.70% (*n* = 4,681) received allopurinol, 0.06% (*n* = 4) febuxostat, 0.05% (*n* = 3) probenecid, and 1.5% (*n* = 89) benzbromarone. Dosage of those medications was not documented.

### 3.3. Reasons for Treatment Initiation with Febuxostat

The standardized patient documentation form assessed reasons for the initiation of febuxostat (insufficient efficacy of previous treatment, compliance issues or interactions with concomitant medication; multiple reasons could be given). The main reason for the treating physician to initiate treatment with febuxostat was insufficient efficacy of the previous ULT (75.1%). In addition, compliance issues with previous treatment (26.4%) and interactions with concomitant medications (10.5%) were documented. Due to the given structure of the questionnaire other reasons or more specified information for the initiation of febuxostat were not captured.

### 3.4. Study Medication, Concomitant Medication, and General Measures

At study start, febuxostat was administered at a daily dose of 80 mg and 120 mg in 87.0% (*n* = 5,175) and 12.0% (*n* = 715) of patients, respectively (1.0% not specified). The prescribed initial daily dose was maintained in most cases (94.0%). Dose increases to 120 mg/day occurred in 4.0% of patients. In 1.0%, the dosage was decreased to 80 mg/day.

Treatment-associated measures included nutrition counseling (84.2%) to achieve a dietary change (78.6%) in addition to weight reduction (62.6%). About half the patients (43.1%) received medical therapy for gout flare prophylaxis in addition to treatment with febuxostat. Diclofenac (24.6%) and ibuprofen (11.4%) were prescribed most frequently. Colchicine was prescribed to a lesser extent (8.7%) and corticosteroids (e.g., prednisolone 2.7%) only in exceptional cases. In consequence of preexisting comorbidities, 76.7% of patients received a mean number of three concomitant drugs. Antihypertensive drugs (e.g., the ACE inhibitor ramipril, 19.9%), lipid-lowering agents such as simvastatin (20.2%), and/or antidiabetic drugs such as metformin (13.4%) were documented most frequently.

### 3.5. Decrease of Uric Acid Levels with Febuxostat

**Figure 1:**
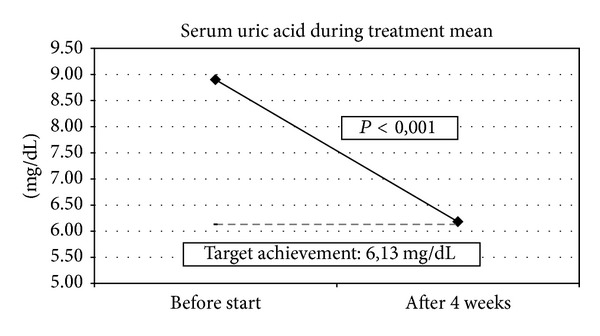
Decrease in serum uric acid levels during the 4-week treatment course with febuxostat.

**Figure 2:**
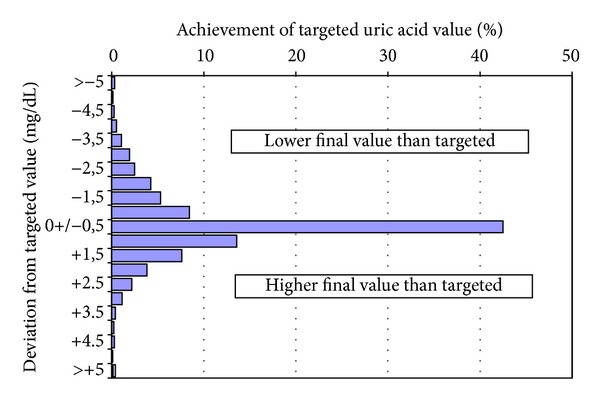
Percentage of patients who reached the individual target value of 6 mg/dL (0 ± 0.5 mg/dL), respectively, who had lower (−1 to >−5 mg/dL) or higher (+1 to >+5 mg/dL) uric acid values. Note that about one-third (29.6%) failed to reach the guideline recommended target [6, 8].

Mean serum uric acid levels decreased significantly from 8.9 ± 1.9 mg/dL (median 8.7 mg/dL; 534.0 *μ*mol/L ± 114.0; median 522.0 *μ*mol/L) at baseline to 6.2 ± 2.5 mg/dL (median 6.2 mg/dL; 372.0 *μ*mol/L ± 150.0; median 372.0 *μ*mol/L) at the final study visit (*P* < 0.001) (Figure 1). This corresponds to a mean decrease of 2.7 ± 2.8 mg/dL (median 2.4 mg/dL; 162.0 ± 168.0 *μ*mol/L; median 144.0 *μ*mol/L) during febuxostat treatment. Physicians documented a mean target value of 6.1 ± 1.0 mg/dL (median 6.0 mg/dL; 366.0 ± 60.0 *μ*mol/L; median 360.0 *μ*mol). 42.5% of patients reached their individual target with an accuracy of ±0.5 mg/dL. 24.7% even surpassed the target by 1 mg/dL to 5 mg/dL. 29.6% failed to reach the target, whereby 13.6% achieved values of 1 mg/dL above the target (Figure 2).

### 3.6. Physicians' Global Assessment and Future Treatment Plan

The vast majority of physicians rated the treatment with febuxostat as very good (68.1%) or good (27.2%).

Febuxostat was planned to be continued in 85.6% of patients, mostly at a daily dose of 80 mg (74.1%).

### 3.7. Safety

In total, 178 adverse events (AE) were documented in 152 patients (2.6%), whereby 134 patients experienced one AE, 12 patients two, 5 patients three, and one patient 5 AE. Adverse events were rated as serious in 15 patients. 127 AE (71.4%) resolved by the end of the observation period and one undesirable effect (0.6%) did not completely resolve. The outcome of 36 AE (20.2%) not related to febuxostat treatment was unknown. In two patients, AE (gout flare and stomach pain with dyspepsia) had not been resolved at the time of reporting. Four fatal events (2.3% of all AE) were reported but were not considered related to the administration of febuxostat. The documented cause of death was cancer progression (*n* = 2), suicide, and cardiac failure, respectively. Among the 178 AE there were 105 gout flares (58.4% of all AE). Skin reactions were reported in 7 patients. Those were pruritus and allergic reaction (e.g., eczema).

254 patients (4.3%) discontinued febuxostat prematurely. Of those, 102 patients (1.7%) did not specify a reason. Poor compliance (1.3%) and AE (0.6%) were the most common reported reasons for discontinuation. Only 0.4% of patients switched treatment, in most cases to allopurinol.

## 4. Discussion

The observational study FORTE evaluated the efficacy and safety of urate lowering treatment with febuxostat in patients not responding to former ULT, patients with adverse events, and patients with other reasons under “real life” conditions in primary care.

### 4.1. Decrease in Serum Uric Acid Levels

At baseline, patients had a high uric acid value of mean 8.9 mg/dL (534.0 *μ*mol/L). After 4-week treatment period with febuxostat, about two-third of patients (67.2%) achieved a significant decrease of serum uric acid (SUA) levels to mean 6.2 mg/dL (372.0 *μ*mol/L). This significant decrease in SUA levels confirms the very potent urate lowering effect of febuxostat documented in the former RCT's [12, 13].

In the study about one-third of patients (29.6%) failed to reach the guideline recommended target of 6.0 mg/dL (360.0 *μ*mol/L), but a dose increase from 80 mg to 120 mg was exerted only in 4.0% of patients. One may argue that one important reason might be the short observation period which does not allow effective titration. But, on the other hand, continued prescriptions beyond the study end indicate that an increase of the daily dosage was not planned. 85.6% of patients continued to receive febuxostat, however, mainly in the starting daily dosage of 80 mg (74.1%).

In the standardized documentation form the treating physician was asked for his personal targeted serum uric acid (SUA) level. Interestingly, we observed a variance of targeted SUA levels between different physicians. The variance of documented target values was ±1.0 mg/dL (±60.0 *μ*mol/L) and the mean individual target value was 6.1 mg/dL (366.0 *μ*mol/L). One reason for uncertainty about the target may be the consequence of varying norm values with broad variance provided by different laboratories. (Furthermore, separate provided norm values for men and women make it even more complicated.) The lack of German guidelines for diagnosis and treatment of gout/symptomatic hyperuricaemia may also be considered as a factor contributing to the uncertainty [7]. At that time as the study was performed recommendations from rheumatologic societies were published (British Society of Rheumatology, EULAR), which recommend “treat gout to target” of at least 6.0 mg/dL (360 *μ*mol/L). These evidence based recommendations seemed obviously not known in German primary care practices   [6, 8]. More recent observations coming from other countries named the nonimplementation of recommendations as one issue for failure of successful gout treatment   [14].

Unawareness of existing recommendations might also be one reason for the observation that less than half the patients (43.1%) received the recommended gout flare prophylaxis in addition to urate lowering treatment. NSAIDs (especially diclofenac and ibuprofen) were administered most frequently while colchicine or prednisolone were used rarely which is in line with other observations [15]. Interestingly, only 125 patients (2.1%) experienced gout flares during the observation period. Reasons for the lack of coadministered gout flare prophylaxis were not collected in the documentation form.

### 4.2. Comorbidities and Concomitant Medication

Gout patients are known to exhibit other comorbidities quite frequently, particularly metabolic disorders [3, 15]. In concordance with other observations, in the present study more than two third of the participants (75.5%) had at least one concomitant disease. This was consistent with the number of patients receiving at least one concomitant drug in addition to febuxostat (76.7%). Avoidance of drug interactions was documented as the third most frequent reason for febuxostat prescriptions (10.5%) in this study. In the context of multiple comedication febuxostat may be preferred as—compared to other urate-lowering drugs—febuxostat does not require dose adjustments when administered in combination with colchicine, NSAIDs (e.g., naproxen and indomethacin), hydrochlorothiazide, warfarin, and desipramine (CYP2D6 substrate). No significant change in *C*_max⁡_ and AUC was observed in patients with mild or moderate liver impairment compared to those with normal liver function after multiple doses of 80 mg febuxostat [12, 16].

The association between gout and impaired renal function is well established and in post-hoc analyses febuxostat has shown to be effective and beneficial in those patients [17]. In this observational study 16.4% of patients were documented to have impaired renal function (eGFR < 89 mL/min). No febuxostat dose adjustments is required in patients with mild to moderate renal impairment (eGFR > 30 mL/min) or in the elderly [11]. In contrast, the daily dose of allopurinol has to be adjusted according to the renal function.

In the first evaluation of the study the physicians were asked for the reasons of starting ULT with febuxostat. Interestingly, the majority of the treating physician indicated insufficient efficacy of the previous ULT (75.1%) which was predominantly allopurinol. Doses of previously administered allopurinol were not assessed. Generally, in Germany, primary care practices the administered doses of allopurinol ranges from 100 to 300 mg daily, usually not exceeding 300 mg daily [1]. This could be partly explained by lacking of safety of higher doses of allopurinol in the long term. Furthermore, potentially worse systemic reactions (AHS, DRESS, etc.) are reported in the literature, especially in patients with renal impairment and multiple comedication [18].

### 4.3. Lifestyle Change

An interesting observation coming from the study was the surprising high percentage of physicians which introduced additional nonpharmacological interventions. The majority of patients were subjected to measures aiming at lifestyle changes. Physicians recommended nutritional counseling (84.2%), dietary changes (78.6%), and/or weight reduction (62.6%). These general measures are significant in managing a “disease of affluence” such as gout. In this context a personal missing communication has been shown to be a very relevant reason for treatment-failure in gout [14]. A nonpharmacological approach might help the patient to get more involved in the disease management [19, 20].

### 4.4. Safety and Tolerability

Altogether, data from the present study confirm that febuxostat is safe and well tolerated in the daily clinical practice, which is in line with the results from pivotal studies [11, 13, 16]. A total of 178 AE (in 152 patients) was documented in the total study population (*n* = 5, 592). 105 AE were gout flares. It could be speculated that some of gout flares could be avoided if concomitant prophylaxis was administered as recommended by guidelines [6, 8, 10]. Nonspecific skin reactions (e.g., pruritus, eczema) were reported in only seven patients. No severe hypersensitivity reactions occurred.

Four fatal events (2 patients died due to cancer progression, one patient due to suicide, and one patient due to cardiac failure) were not related to febuxostat to the best of our knowledge.

Overall, less than 1% of the total patient population discontinued febuxostat prematurely due to any AE. Furthermore, the good compliance confirms that febuxostat was generally well tolerated: 82.0% of patients stated regular administration of the product.

### 4.5. Limitations of the Study

Due to the design of the study and absence of a comparative cohort (e.g., patients treated with allopurinol), the presented data are exclusively of descriptive nature. The diagnosis of gout in all the patients was based in each participating physician judgment; there was no specific information given about the diagnostic certainty, for example, crystal proven gout. As the intention of the study was mainly to assess the safety of febuxostat in daily routine many other aspects and information of the decision making physician was not asked by the standardized documentation form. Due to the given structure of the questionnaire, there were no more detailed reasons for changing to febuxostat captured. In particular, dosage of the previous administered allopurinol was not captured. Furthermore, possible other measures of lack of efficacy of previous ULT, adverse events, and specified information about the interactions with other comedication were not assessed. Reasons for absence of gout flare prophylaxis were not asked. Furthermore, the short 4 weeks follow-up possibly not allowed dose titration of febuxostat which limited further conclusions.

## 5. Conclusion

The present study confirms the results of the pivotal studies regarding the safety and effectiveness of febuxostat in decreasing serum uric acid levels. Accordingly, febuxostat lowers serum uric acid levels also in routine clinical practice from mean values above 8 mg/dL to approximately 6 mg/dL within 4 weeks. The vast majority of patients reached their individual target value or achieved even lower serum uric acid levels, already with the lower dose of 80 mg febuxostat. However, physicians pursued treatment recommendations targeting values < 6 mg/dL (360 *μ*mol/L) inconsistently. Overall, treatment with febuxostat in both available doses (80 mg and 120 mg) was safe and well tolerated in patients with symptomatic hyperuricaemia.

## Figures and Tables

**Table 1:** Demographic and clinical data (*n* = 5,948).

Age (mean ± SD)	63.7 ± 12.6
Gender	*n* (%)
Male	4 318 (73%)
Female	1 561 (26%)
No data available	69 (1%)
Gout flares/year (mean ± SD)	3 ± 2.3
Number of patients with	*n* (%)
Primary gout	4 008 (67%)
Secondary gout	1 278 (21%)
No data available	662 (11%)
Number of patients with	*n* (%)
Gout in the big toe joint	4 138 (70%)
Tophi	540 (9%)
Joint damage/changes∗	1 077 (18%)
Number^1^ of patients with concomitant disease	*n* (%)
With at least one	4489 (75.5%)
Hypertension	4102 (69%)
Hyperlipidemia	2557 (43%)
Diabetes mellitus	1902 (32%)
Impaired renal function	976 (16.4%)
Thyroid dysfunction	431 (7.3%)
Depressions	418 (7%)
History of kidney stones	195 (3.3%)
Other	854 (14.4%)

^1^Percentual reference to number of total collective *n* = 5948.

*Assessment by the treating physician which covers clinical and probably radiographic findings.

Multiple entries per patient possible.
